# Two decades of nonfatal injury data: a scoping review of the National Electronic Injury Surveillance System-All Injury Program, 2001–2021

**DOI:** 10.1186/s40621-023-00455-4

**Published:** 2023-09-07

**Authors:** Livia Navon, Li Hui Chen, Mary Cowhig, Amy Funk Wolkin

**Affiliations:** 1 National Center for Injury Prevention and Control, Centers for Disease Control and Prevention, 4770 Buford Highway, N.E., Mailstop S106-8, Atlanta, GA 30341-3717 USA; 2 Division of Hazard and Injury Data Systems, U.S. Consumer Product Safety Commission, Bethesda, MD USA; 3 Formerly with the Division of Hazard and Injury Data Systems, U.S. Consumer Product Safety Commission, Bethesda, MD USA

**Keywords:** Injury surveillance, Emergency department visits, Epidemiology, Scoping review, NEISS-AIP

## Abstract

**Background:**

Injury is a leading cause of preventable morbidity and mortality in the USA. Ongoing surveillance is needed to understand changing injury patterns to effectively target prevention efforts. Launched jointly in 2000 by the Consumer Product Safety Commission (CPSC) and the Centers for Disease Control and Prevention (CDC), the National Electronic Injury Surveillance System-All Injury Program (NEISS-AIP) provides national-level estimates of US emergency department visits for nonfatal injuries. A scoping review of peer-reviewed articles was conducted to characterize how NEISS-AIP data have been used for injury surveillance in the USA.

**Main Body:**

This review was conducted in accordance with the Preferred Reporting Items for Systematic Reviews and Meta-Analyses (PRISMA) guidelines. Three bibliographic databases (PubMed, Scopus, and Google Scholar) were systematically searched for English language peer-reviewed articles that used NEISS-AIP data as the primary data source during 2001–2021. Key article characteristics from included articles were abstracted to generate descriptive summary statistics to understand the use and limitations of NEISS-AIP for injury surveillance. Database queries returned 6944 citations; 594 citations were manually reviewed, and 167 non-duplicate journal articles were identified. An average of 8.0 articles (range: 1–14) were published annually during 2001–2021. Articles appeared in 72 different journals representing a diverse audience with the majority of articles written by CDC authors. Starting in 2013, a higher proportion of articles were published by non-CDC authors. The largest number of articles examined injury among all age groups (*n* = 71); however, the pediatric population was the specific age group of greatest interest (*n* = 48), followed by older adults (*n* = 23). Falls (*n* = 20) and motor-vehicle-related injuries (*n* = 10) were the most studied injury mechanisms. The most commonly identified limitation identified by authors of reviewed articles was that NEISS-AIP only produces national estimates and therefore, cannot be used for state- or county-level injury surveillance (*n* = 38).

**Conclusions:**

NEISS-AIP has contributed to nonfatal injury surveillance in the USA. CDC and CPSC continue to work together to expand and enhance NEISS-AIP data collection. Researchers are encouraged to continue using this publicly available dataset for injury surveillance.

**Supplementary Information:**

The online version contains supplementary material available at 10.1186/s40621-023-00455-4.

## Background

In the USA, injury is the leading cause of death for persons less than 45 years of age (Centers for Disease Control and Prevention (CDC) 2022) and causes significant morbidity among all age groups, with injury- and poisoning-related visits accounting for the highest proportion of all treat-and-release emergency department (ED) visits (Weiss and Jiang 2021). To monitor and understand injury patterns, several surveillance data systems are utilized (Horan and Mallonee 2003; Institute of Medicine (IOM) 1999). A key system for understanding nonfatal injuries in the USA is the National Electronic Injury Surveillance System (NEISS) which monitors ED visits for injuries involving consumer products (Consumer Product Safety Commission (CPSC) 2022). NEISS data collection began in 1971 and has been maintained by the CPSC since 1973 (CPSC 2022).

While CPSC has jurisdiction over some 15,000 types of products, injuries due to specific consumer products such as alcohol, motor vehicles, drugs, and firearms fall outside CPSC jurisdiction and therefore are not systematically collected in NEISS. Additionally, injuries due to non-consumer products (e.g., certain types of occupational injuries), injuries where no product is mentioned (e.g., “fell to ground”), or intentionally inflicted injuries (e.g., assaults and injuries due to self-harm) are not included in NEISS (CPSC 2022). In 1997, a pilot study by CPSC and the CDC to test expansion of NEISS data collection to include ED visits for all-cause nonfatal injuries was successful (IOM 1999; Schroeder and Ault 2001). In 1999, the IOM Committee on Injury Prevention and Control recommended that NEISS data collection be permanently broadened in order to provide “a new and important tool for gathering national estimates and monitoring national trends in injury morbidity, for identifying emerging problems, for evaluating interventions through follow-up studies, and for providing data for policy decisions” (IOM 1999). In July 2000, CPSC and CDC launched the NEISS-All Injury Program (NEISS-AIP) surveillance system to collect ED data on all-cause nonfatal injury from a subset of hospitals that report NEISS data (CDC, 2001). Data collection continues through the present with both NEISS and NEISS-AIP data released annually.

NEISS was designed to collect data from a stratified probability sample of approximately 100 US hospitals that serve the general population, have at least six inpatient beds, and provide 24-h ED services (CDC 2022). Hospitals sampled include very large metropolitan hospitals with trauma centers, as well as urban, suburban, rural, and children's hospitals. NEISS-AIP data have historically been collected at a two-thirds subset of sampled NEISS hospitals. The number of NEISS and NEISS-AIP hospitals has varied over time as sampled hospitals closed or changes in ED services resulted in certain hospitals becoming ineligible for sample inclusion.

NEISS-AIP collects data on nonfatal injury visits based on the patient’s vital status upon arrival to the ED. Only first-time injuries are included in the sampling frame; therefore, visits by persons seeking additional ED care for a previously sustained injury are excluded. NEISS-AIP uses CPSC-trained coders within each sampled hospital to abstract and code data directly from medical records for eligible nonfatal injury-related ED visits. Coded data and a brief summary narrative are transmitted electronically to CPSC, where quality assurance coders review the data for accuracy and assign the precipitating and immediate mechanism (cause) of injury. Historically, NEISS-AIP has collected data from about 500,000 injury-related ED visits annually. These data are weighted to produce national estimates of the approximately 30 million US nonfatal injury-related ED visits that occur each year.

Data elements currently available in NEISS-AIP include treatment date, patient demographic information (e.g., age, sex, race/ethnicity), the mechanism of injury (e.g., a fall, motor vehicle occupant), the injury intent (e.g., unintentional, assault), up to two visit diagnoses (e.g., fracture, anoxia), up to two injured body parts (e.g., head, neck), the type of location where the injury occurred (e.g., home, street/highway), products involved in the injury (e.g., toy, bicycle), whether the injury was related to participating in sports or work, the patient’s disposition (e.g., treated and released, admitted), and an open text narrative field. To complete the narrative, data coders are instructed to review all available ED notes and summarize pertinent information, including a description of what the patient was doing when the incident occurred (i.e., the sequence of events), injury intent, the product(s) involved, and the location where the incident occurred. When available, information about the use (or lack of) protective equipment is included in the narrative (Inter-university Consortium for Political and Social Research (ICPSR) 2022).

NEISS-AIP data are publicly available and can be queried through CDC’s Web-Based Injury Statistics Query and Reporting System (WISQARS) (CDC 2022) or downloaded for analysis (ICPSR 2022). To understand how NEISS-AIP data have been used for injury surveillance, CDC and CPSC authors conducted a literature review of peer-reviewed journal articles published from January 2001 to December 2021 that used NEISS-AIP as a primary data source. The purpose of this scoping review was to understand how NEISS-AIP data have contributed to injury surveillance, to identify the types of researchers who have utilized these data, where findings were published, and to highlight populations and injury topics studied. Given the expansion of electronic health record use since NEISS-AIP was initiated (Office of the National Coordinator for Health Information Technology 2023), the study goals also included determining whether NEISS-AIP data continue to be used and the limitations of use for nonfatal injury surveillance.

## Methods

### Literature search and information sources

A systematic literature review was conducted in accordance with the Preferred Reporting Items for Systematic reviews and Meta-Analyses (PRISMA) guidelines (Page et al. 2021). To identify journal articles that used NEISS-AIP data for original research during 2001–2021, three bibliographic databases (PubMed, Scopus, and Google Scholar) were systematically searched for English language peer-reviewed articles. Query terms used for each database are shown in Table 1 and included terms such as “NEISS-AIP” and “WISQARS and nonfatal.” The specified query terms were used to search article titles, abstracts, and key words (Scopus) or all available search fields (PubMed and Google Scholar).

**Table 1:** Systematic literature review query terms and citations identified by bibliographic database, NEISS-AIP articles, 2001–2021

Bibliographic database	Search query terms	Date query executed	Number of citations	Citations manually reviewed	Included citations	Non-duplicate included citations
PubMed	((NEISS AIP) OR (NEISS-AIP)) OR (“all injury program”) or ((web-based injury and statistics query reporting system) and (nonfatal)) or ((web-based injury and statistics query reporting system) and (“non fatal”)) or ((web-based injury and statistics query reporting system) and (non-fatal)) or (National Electronic Injury Surveillance System-All Injury Program) or ((WISQARS) and (nonfatal)) or ((WISQARS) and (non-fatal)) or ((WISQARS) and (“non fatal”)) AND (2000:2021[pdat])	1/26/2022	140	140	124	124
Scopus	TITLE-ABS-KEY (“NEISS AIP” OR NEISS?AIP OR NEISS*AIP OR (all injury program) OR ( wisqars AND non-fatal) OR ( wisqars AND “non fatal”) OR ( wisqars AND nonfatal) OR”( web*based injury AND statistics query reporting system) AND nonfatal) OR (( web-based injury AND statistics query reporting system) AND non-fatal) OR ((web-based injury AND statistics query reporting system) AND “non fatal”) OR ((web-based injury AND statistics query reporting system) AND “nonfatal”)) AND ( EXCLUDE ( SR “TYPE,"b"”)) AND ( EXCLUDE ( PUBY “AR,2022)”)	1/27/2022	104	104	91	5
Google Scholar	NEISS AIP	1/27/2022	1490^a^	270	137	31
Google Scholar	WISQARS AND (nonfatal OR “non fatal” OR non-fatal)	1/31/2022	5210^b^	80	21	7
Total	–	–	6944	594	373	167

^a^Google Scholar does not provide the ability to exclude non-article citations. Additionally, multiple duplicates are included in search results. The first 270 Google Scholar results were evaluated; between results 260 and 270, no further citations were added and relevance of results to the search declined. In the first 270 reviewed results, 12 book references, 9 conference abstracts, 8 publications where the author’s name matched the search term, 6 correspondence letters, 5 incorrect links, 4 white papers, 4 research summaries, 3 student theses, 1 article in Spanish, 1 website, 1 literature review, and 1 newsletter were excluded (total = 53/270 reviewed citations (20%))

^b^The first 80 Google Scholar results were evaluated; between results number 70 and 80 no further citations were added, and relevance of results to the search declined. In the first 80 reviewed results, 6 legal journal publications, 4 book references, 2 white papers, 2 conference publications, 1 factsheet, 1 conference abstract, 1 commentary, 1 foreign language citation, 1 factsheet, 1 citation that could not be accessed, 1 dissertation, and 1 meta-analysis were excluded (total = 22/80 reviewed citations (28%))

Citations identified by querying the three bibliographic databases were evaluated in sequence beginning with PubMed, followed by Scopus, and lastly, Google Scholar. Google Scholar provides limited ability to filter query results, includes citations for gray literature such as conference proceedings and white papers, and returns a higher proportion of duplicate citations than other bibliographic databases (Haddaway et al. 2015). Additionally, Google Scholar citations are ordered by a proprietary relevance score that is not displayed with the query results (Rovira et al. 2019). Given the limited ability to specify query terms, two separate queries with broad search terms were used to query Google Scholar. Due to the large volume of Google Scholar citations identified, after consultation with a CDC librarian, for each separate Google Scholar query, citations were evaluated until ten consecutive, non-relevant query results were identified. Subsequent Google Scholar query results were not evaluated for inclusion.

### Eligibility criteria

Journal articles were included in this review only if NEISS-AIP data were analyzed to present original research findings. Articles were excluded if NEISS-AIP data were presented for context only (e.g., one or two injury statistics in the background section of the article). Articles were also excluded if the use of NEISS-AIP data was stated but the methods described and data analyses conducted either (a) indicated that only NEISS data were used for analysis or (b) it could not be determined whether the data used were from NEISS or NEISS-AIP. Lastly, articles were excluded if data from special studies based on NEISS-AIP, such as the NEISS-Cooperative Adverse Drug Event Surveillance project (NEISS-CADES) (Jhung et al. 2007), were used, and the article did not also use NEISS-AIP as a primary analytic dataset.

Abstracts for all relevant citations identified in the three different databases were evaluated by one reviewer (LN) to determine whether the inclusion criteria were met. If sufficient data were not available in the abstract or the data source used was unclear, the full article was reviewed. If based on the description of the study methodology it was still unclear whether the article should be included in this review, the full article was also reviewed by a second author (LC or MC) and the final inclusion determination was based on discussion and agreement among all authors.

For included articles, key article characteristics were abstracted into a spreadsheet and used to generate descriptive summary statistics. Abstracted parameters included bibliographic information; lead author affiliation (CDC/non-CDC); the number of years of NEISS-AIP data used for analysis; whether additional data sources were used in the manuscript; the study population; key injury data, including the type of injury, injury mechanism, and injury intent studied; NEISS-AIP limitations outlined in the article; and the number of times each article was cited in either an original research article or a review article. Citation statistics were abstracted from Scopus (Elsevier 2022) in July 2022. If articles were not indexed in Scopus, citation statistics from PubMed (National Library of Medicine 2022) were used.

## Results

**Fig. 1:**
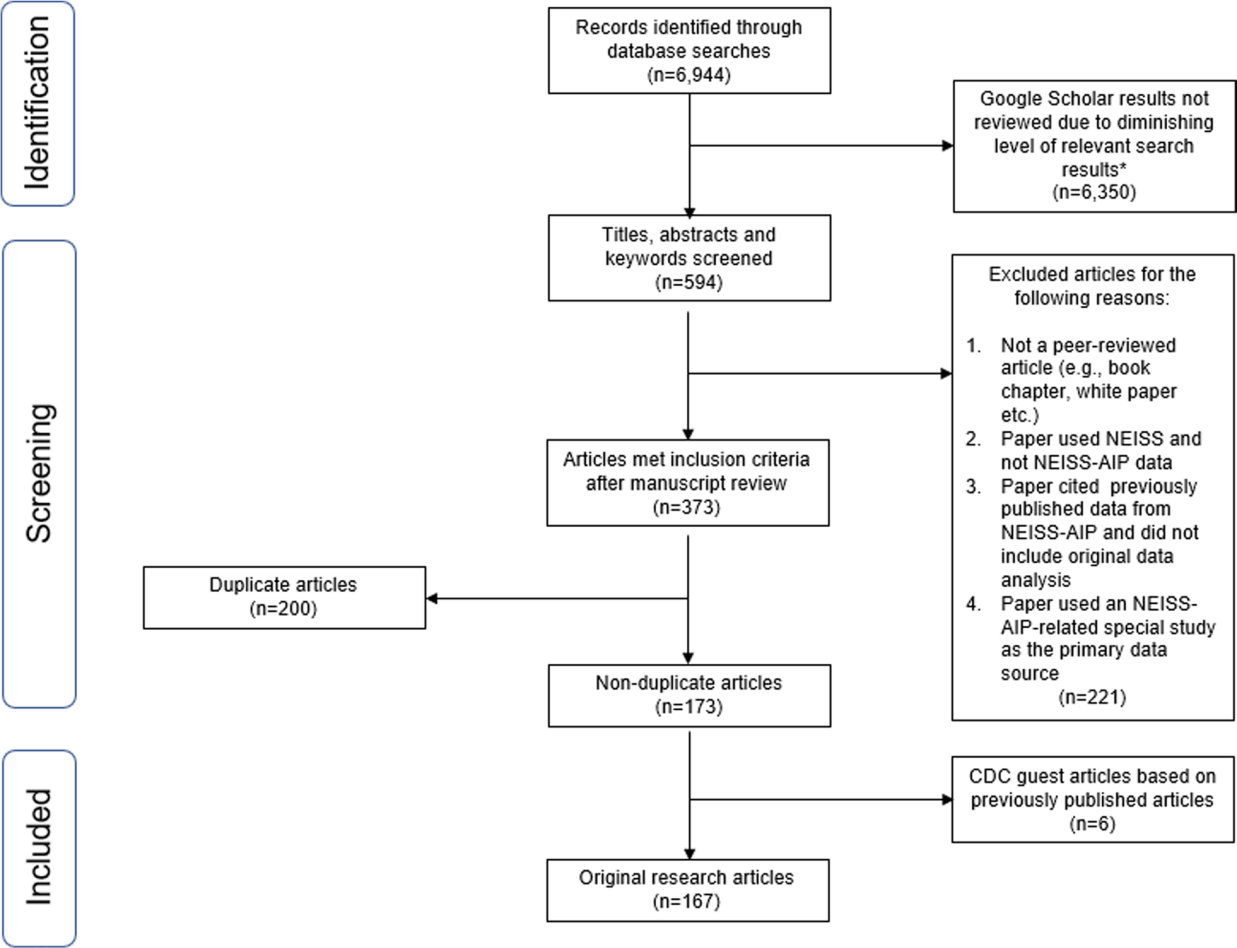
Systematic literature review summary results, NEISS-AIP articles, 2001–2021

A total of 6944 publication citations were identified across the three bibliographic databases (Table 1 and Fig. 1). The largest number of citations was identified using Google Scholar based on two separate queries. However, 6350 citations were removed after ten consecutive non-relevant results were identified within each Google Scholar search. A total of 594 citations were manually reviewed across all three databases and 173 non-duplicate journal articles identified. Six articles based on the same NEISS-AIP primary analysis but published in two different journals were only included once. In total, 167 journal articles met the inclusion criteria for this review. The full list of articles is available in Additional file 1 [see all articles included in the NEISS-AIP Scoping Review].

**Fig. 2:**
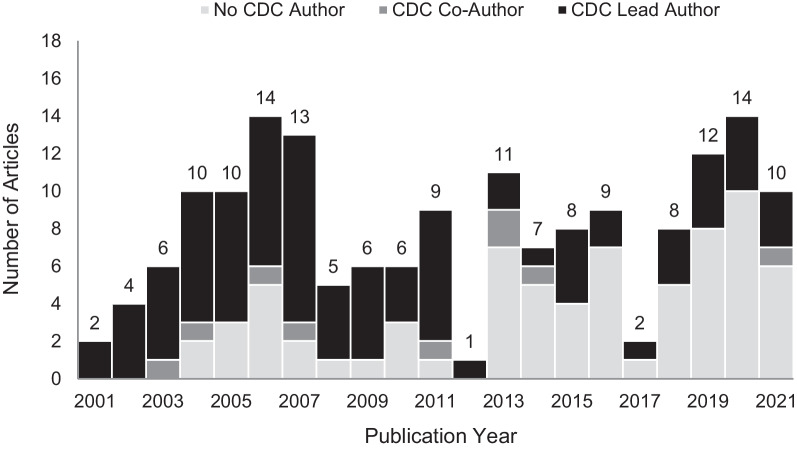
Number of NEISS-AIP articles by publication year and author affiliation, 2001–2021 (*n* = 167)

During 2001–2021, an average of 8.0 articles (range: 1–14 articles) that used NEISS-AIP data as a primary data source were published annually (Fig. 2). Most articles (57%) included a CDC-affiliated author as either the lead author (91%) or a co-author (9%). During the initial years of NEISS-AIP data collection (2001–2003), all articles had a CDC author; in subsequent years, articles were increasingly published by non-CDC authors. Non-CDC authors were primarily affiliated with universities and academic medical institutions. Starting in 2013, in most years, there were more publications by non-CDC authors than CDC authors (Fig. 2).

Assessing diversity in dissemination and audience, the 167 reviewed articles appeared in 72 different journals. CDC’s “Morbidity and Mortality Weekly Report” (MMWR) was the journal with the most articles (*n* = 37), followed by “Injury Prevention” (*n* = 16), the “Journal of Safety Research” (*n* = 8), and “Pediatrics” (*n* = 8). Of the 72 journals, 52 journals published only one NEISS-AIP article during 2001–2021. These journals were often subject matter-specific journals such as “Brain Injury,” which published on non-fatal traumatic brain injuries (TBIs) among children and adolescents (Ali et al. 2019), “Traffic Injury Prevention,” which published on traffic-related injuries (Naumann et al. 2010), and “Journal of Studies on Alcohol,” which published on alcohol-related injuries (Elder et al. 2004).

Because NEISS-AIP data have been collected for more than twenty years, it is possible to utilize multiple years of data for analysis. In addition to monitoring injury trends, multiple years of data can be combined to assess ED visits for less common injuries (e.g., drowning) with stable injury estimates, as well as to be able to stratify by patient demographics. Most articles (72%) used more than one year of NEISS-AIP data for analysis: 35% used 2–5 years of data, 22% used 6–10 years of data, and 16% used more than ten years of data. Many of the publications that used more than ten years of data examined trends in injury-related ED visits over time (Coronado et al. 2015; Mercado et al. 2017; Waltzman et al. 2020; Orces and Orces 2020; Olufajo et al. 2021).

Nonfatal ED visit data provide useful insight into injury incidence; however, understanding the broader burden of injury requires additional sources of data. Although most reviewed publications (65%) used NEISS-AIP data as the sole data source, nonfatal ED data were commonly presented with other injury-related data to better understand a particular aspect of injury epidemiology. For example, some authors included mortality data to estimate the total burden of fatal and nonfatal injuries (Ballesteros et al. 2003; Gilchrist et al. 2004; Vyrostek et al. 2004). Others presented data from national surveys such as the Behavioral Risk Factor Surveillance System (CDC, 2011) or the Youth Risk Behavioral Surveillance System (Sumner et al. 2015) to understand injury-associated behaviors. NEISS-AIP data were also used in conjunction with other data sources to estimate injury-related costs such as lifetime medical cost (Corso et al. 2006) and productivity loss (Stevens et al. 2006).

Age is a key risk factor for many injuries. NEISS-AIP collects data on all ages, and both pediatric and general hospitals are included in the sampling frame. The majority of articles used NEISS-AIP data to understand injury-related ED visits in a particular age group, to look at ED visits for a particular type of injury, or to examine both factors together (Table 1). The largest proportion of articles included data on all ages (43%); however, injury among the pediatric population (29%) accounted for a large share of articles. Fourteen percent (14%) of articles examined injury among older adults. Most of these articles defined older adults as persons 65 years of age and older, though some studies focused on other age categories (e.g., adults aged 60 years and older) (Khurana and Loder 2021; Logan et al. 2019; Orces and Martinez 2011). Most articles (79%) presented data on all nonfatal injuries, rather than focusing on a particular type of injury. However, some authors were interested in understanding specific injuries such as TBI/head trauma (8%) or fractures/dislocations (4%). ED visits for TBI/head trauma among the pediatric population were of particular interest, accounting for 69% (9 of 13 articles) of TBI/head trauma-related articles (Table 2).

**Table 2:** Number of NEISS-AIP articles by type of injury and age group studied, 2001–2021 (*n* = 167)

Type of injury	Age group					
	All ages	Pediatric^a^	Older adult^b^	Other age group^c^	Adults^d^	Grand total
All injuries	56	36	20	16	4	132
TBI^e^/head trauma	4	9	–^f^	–	–	13
Fractures/dislocations	1	1	2	2	–	6
Poisoning	4	1	–	–	–	5
Eye injuries	2	1	–	–	–	3
Submersion	2	–	–	–	–	2
Burn	–	–	1	–	1	2
Other^g^	1	–	–	–	1	2
Craniofacial injuries	–	–	–	1	–	1
Finger amputation	1	–	–	–	–	1
Grand total	71	48	23	19	6	167

^a^Articles that limited analyses to persons < 20 years. Some articles looked at subsets of this age group, for example, children ≤ 4 years (Haarbauer-Krupa et al. 2019)

^b^Articles that limited analyses to persons ≥ 50 years

^c^Articles that analyzed data for age groups that cross population categories, for example, injuries among persons aged 10–70 years (Friedmann and Kohn 2004) or 15–54 years (Khurana et al. 2021)

^d^Articles that limited analyses to persons ≥ 18 years or persons ≥ 20 years

^e^TBI = Traumatic brain injury

^f^Zero articles found in category

^g^One article that looked at TBI/head trauma and other fractures together (Wei and Hester 2014) and one article that examined traumatic nonfatal injuries (Shults et al. 2009)

Identifying the intent of the injury, that is, whether the injury was inflicted purposefully and by whom [self or another person] or unintentionally is an important component of understanding injury patterns (CDC 2022). Additionally, injuries can be characterized by the mechanism of injury, which is the way a person sustained an injury, such as a dog bite or motor vehicle accident (CDC 2022). Most articles did not focus on a specific injury mechanism and instead were interested in ED visits for all injury mechanisms combined (*n* = 96) (Table 3). The most commonly examined injury mechanisms were falls (*n* = 20) and motor vehicle crashes (i.e., injury to a motor vehicle occupant resulting from a collision, rollover, boarding, alighting, or some other event involving a vehicle, object, or pedestrian) (*n* = 10). Similarly, most articles did not focus on a specific intent, but rather, reported on all injuries, regardless of intent (*n* = 69). When an intent was specified, unintentional (accidental) injuries were most commonly studied (*n* = 62), followed by assault (*n* = 19) and self-harm (*n* = 11) (Table 3).

**Table 3:** Number of NEISS-AIP articles by injury mechanism and injury intent, 2001–2021 (*n* = 167)

Injury mechanism	Injury intent^a^						
	All/not specified^b^	Unintentional	Assault	Self-harm	Legal intervention	Multiple^c^	Total
All/not specified^b^	40	24	18	9	4	1	96
Fall	2	18	–^d^	–	–	–	20
Motor vehicle occupant	6	4	–	–	–	–	10
Other bite, including sting	4	2	–	–	–	–	6
Poisoning	1	4		1	–	–	6
Firearm	4	–	1	–	–	–	5
Dog bite	3	1		–	1	–	5
Pedal cyclist	2	1	–	–	–	–	3
Inhalation/suffocation		3	–	–	–	–	3
Motorcyclist	3		–	–	–	–	3
Pedestrian	1	1	–	–	–	–	2
Drowning/nonfatal submersion	–	2	–	–	–	–	2
Fire/burn	1	–		1			2
Natural/environmental	–	1	–	–	–	–	1
Struck by/against	1	–	–	–	–	–	1
Cut/pierce	–	1	–	–	–	–	1
All transportation	1	–	–	–	–	–	1
Total	69	62	19	11	5	1	167

^a^Injury intent classifies whether an injury was an act carried out on purpose by oneself or by another person(s), with the goal of injuring or killing. Specific types of intent classified in NEISS-AIP and available for analysis are defined as follows (CDC 2022):

*Assault*: Injury from an act of violence where physical force by one or more persons is used with the intent of causing harm, injury, or death to another person; or an intentional poisoning by another person. This category also includes injuries related to sexual assault. *Legal Intervention*: Injury or poisoning caused by police or other legal authorities (including security guards) during law enforcement activities. *Self-Harm*: Injury or poisoning resulting from a deliberate violent act inflicted on oneself with the intent to take one’s own life or with the intent to harm oneself. This category includes suicide, suicide attempt, and other intentional self-harm. *Unintentional*: Injury or poisoning that is not inflicted by deliberate means (i.e., not on purpose). This category includes those injuries and poisonings described as unintended or “accidental,” regardless of whether the injury was inflicted by oneself or by another person. Also, includes injury or poisoning where no indication of intent to harm was documented in the ED record

^b^All injury intents included in the analysis or injury intent not specified; all injury mechanisms or injury mechanism not specified

^c^One article examined both interpersonal violence and self-harm (Corso et al. 2007)

^d^Zero articles found in category

The frequency with which an article is cited is one measure of the publication’s scientific influence. The number of citations of the articles included in this review varied considerably. There were 7370 citations identified for all the included articles with a median of 18 citations (range: 0–875 citations). The three most-cited articles presented data on: (1) the costs of fatal and nonfatal falls among older adults using multiple data sets, including the Medical Expenditure Panel Survey (Stevens et al. 2006) (875 citations); (2) ED visits for adverse drug events using both NEISS-AIP and NEISS-CADES data (Budnitz et al. 2006) (594 citations); and (3) nonfatal ED visits for TBIs among youth related to sports and recreation using only NEISS-AIP data (314 citations) (Gilchrist et al. 2011).

There are limitations when using NEISS-AIP data for injury surveillance (Table 4). The most common limitation identified by authors of articles included in this review was that NEISS-AIP provides only national estimates and therefore state and local injury estimates are not available (*n* = 38). Another commonly identified limitation (*n* = 35) was that NEISS-AIP has incomplete information on protective factors and circumstances (e.g., helmets for bicycling-related injuries). Starting with treatment date January 1, 2019, NEISS-AIP added variables to capture a second injury diagnosis and a second body part injured. Prior to 2019, NEISS-AIP captured only the injury diagnosis and injured body part for the most severe injury sustained by patients. Several authors (*n* = 32) cited this as a limitation and indicated that this may have resulted in an undercount of the injury of interest, as patients can sustain multiple injuries as a result of an injury-causing event. Other commonly cited limitations included that NEISS-AIP does not include outcome information (e.g., disability due to injury) (*n* = 18), information on injury severity (*n* = 13) nor detailed location information (e.g., if an injury occurred in the home, in which room) (*n* = 13).

**Table 4:** NEISS-AIP limitations identified by authors of NEISS-AIP articles, 2001–2021 (*n* = 167)

NEISS-AIP limitation	Number of articles
Only provides national estimates therefore, state and local estimates are not available	38
Doesn’t capture sufficient information on protective factors and injury circumstances	35
Only captures information on most severe injury diagnosis or injured body part*	32
Limited and variable amount of data details captured in narratives	20
No injury outcome information	18
Lack of information on injury severity	13
Missing detailed location information (e.g., room in home where injury occurred)	13
Small sample size can result in unstable estimates for some injuries*	12
No measures of socioeconomic status (e.g., health insurance coverage) or other psycho-social variables (e.g., drug or alcohol use)*	11
High level of missing race/ethnicity data	7
Short (two-line) narrative captures limited information*	5
Limited data available in the ED record (e.g., use of protective equipment) which is used as the source of NEISS-AIP data	4
Body part injured not specific enough as only body region captured	4
Data not linked to other data sources for verification (e.g., police reports)	3
ICD-9-CM codes not captured	3
Unknown and unintentional injury intents grouped together with potential for misclassification	3
Injury intent difficult to identify in emergency department data (e.g., for interpersonal violence and self-inflected injuries)	3
No data on sexual orientation or gender identity**	2
No product codes available for more recently introduced consumer products	1

*Starting with treatment date January 1, 2019, NEISS-AIP added variables to capture a second injury diagnosis, a second body part injured, and whether alcohol or drugs contributed to the injury. Additionally, the maximum length of the narrative text was increased from 142 to 400 characters

**Starting with treatment date January 1, 2021, an additional code for recording patient sex as non-binary was added. This allows the hospital abstractor to report a patient’s sex as it is captured in emergency department records and includes non-binary, intersex, and other designations

## Discussion

This scoping review summarizes how NEISS-AIP data were used for injury research during 2001–2021. The 167 articles included in this review appeared in more than 70 different journals, indicating that research findings based on NEISS-AIP data are reaching a varied audience and reflecting the wide spectrum of injuries that have been studied. CDC’s flagship publication, “MMWR,” accounted for the highest number of articles with these articles primarily authored by CDC staff. The upward trend in in articles written by non-CDC authors in recent years demonstrates the usefulness of these data for external researchers, and it is expected that NEISS-AIP data analyses will continue to be published broadly and to reach a diverse readership. As demonstrated by the articles included in this scoping review, NEISS-AIP provides epidemiologic data that can inform policymakers and public health officials. These data can be used to establish baselines, identify trends, and predict future challenges.

Most articles used more than one year of NEISS-AIP data for analysis. Although NEISS-AIP has historically collected data from more than 500,000 ED visits annually, when studying more granular injury topics, especially in specific age groups, it is often necessary to increase sample size by combining years of data. Limited sample size may explain why most reviewed articles studied ED visits for all injury intents and mechanisms combined, rather than specific injury factors. CDC and CPSC have guidance on which NEISS-AIP estimates can be presented (i.e., data should not be displayed when there are fewer than 20 ED visits (unweighted data), fewer than 1200 ED visits (weighted data) or estimates have a coefficient of variation greater than 30%) (CDC 2022). Detailed NEISS-AIP analytic guidance is available (Schroeder and Ault 2001) to assist researchers.

NEISS-AIP was most often the sole data source in reviewed articles underscoring the utility of these data for injury surveillance. However, NEISS-AIP nonfatal injury data can be examined with other data sources, such as mortality data or cost of injury data, to provide a more complete view of a particular injury topic. NEISS-AIP data, fatal injury data from the National Vital Statistics System and injury cost estimates are all publicly available on CDC’s WISQARS interactive, online injury data query system (CDC 2022).

This scoping review has at least three limitations. First, our review was limited to journal articles that used NEISS-AIP as a primary source of data. While conducting this review, we identified several journal articles that presented NEISS-AIP-derived statistics for context but used a different primary data source for analysis. These articles could not be systematically identified and therefore were not included in this review. Additionally, NEISS-AIP data are used widely in injury-related policy and communications products and have been used to set key national health benchmarks and goals such as Healthy People 2030 (U.S. Department of Health and Human Services 2023). Therefore, the impact of NEISS-AIP on injury epidemiology and policy is underrepresented by the inclusion criteria used for this review. Second, journal articles that used NEISS-AIP-linked special studies were not included in this analysis unless NEISS-AIP was also a primary data source. One of the successes of the NEISS-AIP collection methodology is that it can be leveraged to collect additional data about specific types of injuries such as adverse drug events (Jhung et al. 2007) and injuries due to self-directed violence (Ehlman et al. 2021). However, this review was focused on understanding use of NEISS-AIP data specifically, and therefore, articles that only used data from these special studies were excluded. Third, many of the reviewed articles did not identify the injury intent of NEISS-AIP cases included in analyses, and therefore, for these reviewed articles, the intent studied was classified as all intents/intent not specified. Researchers who use NEISS-AIP data are encouraged to explicitly identify the injury intent examined in their analyses to allow for accurate result interpretation.

In evaluating articles for this review, we excluded six articles where researchers were unclear in explaining whether NEISS or NEISS-AIP was used for analysis. Although both data systems are managed by CPSC, NEISS collects ED visit data only on injuries related to consumer products under CPSC jurisdiction, while NEISS-AIP collects ED visit data for all nonfatal injuries. Additionally, NEISS-AIP has data on injury intent and mechanism that are not available in NEISS. One advantage of NEISS is the larger sample of hospitals used for data collection (approximately 100 hospitals). However, in 2022, NEISS-AIP data collection was expanded to the full NEISS sample of hospitals (Office of Management and Budget 2023). This increased sample size will allow for more precise annual estimates and the ability to better detect changes in patterns of all-cause nonfatal injury.

Even with the increased adoption of electronic health records, NEISS-AIP continues to provide useful data for injury research as evidenced by a similar number of articles published in recent years (> 10 articles per year in 2019–2021) as in the early years of NEISS-AIP data collection (2001–2002). A key strength of NEISS-AIP is the focus on medical record review to capture cases that can be more sensitive than surveillance systems relying only on administrative data (Stanley et al. 2018; Barber et al. 2022). Another key advantage of NEISS-AIP coder abstraction is the open text narrative field which captures contextual and circumstantial information not captured in administrative codes. Although NEISS-AIP diagnosis, intent, and mechanism codes do not align fully with the International Classification of Diseases, 10th Revision, Clinical Modification (ICD-10-CM) (CDC 2023) routinely used for coding healthcare encounters, a limited comparability study of NEISS and International Classification of Diseases, 9th Revision, Clinical Modification injury coding, which preceded the currently used ICD-10-CM coding schema, found high comparability for most injury types (Thompson et al. 2014).

## Conclusions

NEISS-AIP data collection began in the year 2000 with the goal of improving injury surveillance in the USA. Since its inception, NEISS-AIP has led to a better understanding of nonfatal injury in the US population by collecting data on all-cause nonfatal injury-related ED visits. Specific limitations of NEISS-AIP mentioned by authors of articles included in this scoping review have recently been addressed. Beginning in 2019, indicators of alcohol and drug use were added to NEISS-AIP. A variable was also added to capture data on a second injured body part and to capture a second injury diagnosis. Additionally, the length of the narrative was expanded from 142 to 400 characters to capture more detailed information on injury circumstances and protective factors. Beginning in 2021, information on individuals who identify as non-binary, intersex, or other designations began to be systematically collected. Although analysis of NEISS-AIP data by race and ethnicity (which was added as a variable in 2019) is currently limited due to a high proportion of missing data (Office of the Assistant Secretary for Planning and Evaluation 2023), CDC recently published a strategy for imputing these data (Liu et al. 2023).

CDC and CPSC continue to work together to support, expand, and enhance NEISS-AIP data collection. The increased sample size beginning in 2022 will allow researchers to focus on ED visits for specific types of injury mechanisms or intents that may have previously resulted in imprecise or suppressed estimates. Coupled with the additional variables added in 2019 and information on gender identity added in 2021, a more complete picture of nonfatal injury is now possible. Researchers are encouraged to continue using this publicly available dataset for injury surveillance in the USA.

### Supplementary Information

**Additional file 1.** See all articles included in the NEISS-AIP Scoping Review.

## Data Availability

NEISS-AIP data are publicly available from: https://www.icpsr.umich.edu/web/ICPSR/series/198/studies.

## Abbreviations

CDC: Centers of Disease Control and Prevention
CPSC: Consumer Product Safety Commission
ED: Emergency department
ICD: International Classification of Diseases
MMWR: Morbidity and Mortality Weekly Report
NEISS: National Electronic Injury Surveillance System
NEISS-AIP: National Electronic Injury Surveillance System-All Injury Program
NEISS-CADES: National Electronic Injury Surveillance System-Cooperative Adverse Drug Event Surveillance Project
PRISMA: Preferred Reporting Items for Systematic Reviews and Meta-Analyses
TBI: Traumatic brain injury
WISQARS: Web-Based Injury Statistics Query and Reporting System
