# Supplementary material for: Two decades of nonfatal injury data: a scoping review of the National Electronic Injury Surveillance System-All Injury Program, 2001–2021
Source: Inj Epidemiol. 2023 Sep 7;10:44. doi: 10.1186/s40621-023-00455-4 (PMC10486050; doi:10.1186/s40621-023-00455-4)
Supplement: Supplementary file 1 — Additional file 1. See all articles included in the NEISS-AIP Scoping Review. [file 40621_2023_455_MOESM1_ESM.docx]

**Supplementary Material: All Articles Included in the NEISS-AIP Scoping Review**

1. Adams AL, Schiff MA. Childhood soccer injuries treated in U.S. emergency departments. *Acad Emerg Med* 2006;13(5):571-74. doi: 10.1197/j.aem.2005.12.015

2. Ali B, Lawrence B, Miller T, et al. Consumer products contributing to fall injuries in children aged &lt;1 to 19 years treated in US emergency departments, 2010 to 2013: an observational study. *Glob Pediatr Health* 2019;6 doi: 10.1177/2333794X18821941

3. Ali B, Lawrence BA, Miller T, et al. Products and activities associated with non-fatal traumatic brain injuries in children and adolescents - United States 2010-2013. *Brain Inj* 2019;33(11):1425-29. doi: 10.1080/02699052.2019.1631483 [published Online First: 2019/07/30]

4. Amanullah S, Heneghan JA, Steele DW, et al. Emergency department visits resulting from intentional injury in and out of school. *Pediatrics* 2014;133(2):254-61. doi: 10.1542/peds.2013-2155

5. Amanullah S, Schlichting LE, Linakis SW, et al. Emergency department visits owing to intentional and unintentional traumatic brain injury among infants in the United States: a population-based assessment. *J Pediatr* 2018;203:259-65.e1. doi: 10.1016/j.jpeds.2018.08.023

6. and CfDC, Prevention. Vital signs: nonfatal, motor vehicle--occupant injuries (2009) and seat belt use (2008) among adults --- United States. *MMWR Morb Mortal Wkly Rep* 2011;59(51):1681-86.

7. and CfDC, Prevention. Drowning--United States, 2005-2009. *MMWR Morb Mortal Wkly Rep* 2012;61(19):344-7. [published Online First: 2012/05/18]

8. Annest JL, Haileyesus T, Clower J, et al. Nonfatal, unintentional, non--fire-related carbon monoxide exposures--United States, 2004-2006. *MMWR Morb Mortal Wkly Rep* 2008;57(33):896-9. [published Online First: 2008/08/22]

9. Armstrong GW, Chen AJ, Linakis JG, et al. Motor vehicle crash-associated eye injuries presenting to U.S. emergency departments. *West J Emerg Med* 2014;15(6):693-700. doi: 10.5811/westjem.2014.5.20623 [published Online First: 2014/09/24]

10. Armstrong GW, Kim JG, Linakis JG, et al. Pediatric eye injuries presenting to United States emergency departments: 2001-2007. *Graefes Arch Clin Exp Ophthalmol* 2013;251(3):629-36. doi: 10.1007/s00417-011-1917-0

11. Baker SP, Hu G, Wilcox HC, et al. Increase in suicide by hanging/suffocation in the US, 2000–2010. *Am J Prev Med* 2013;44(2):146-49.

12. Bakhos LL, Lockhart GR, Myers R, et al. Emergency department visits for concussion in young child athletes. *Pediatrics* 2010;126(3):e550-e56. doi: 10.1542/peds.2009-3101

13. Ballesteros MF, Schieber RA, Gilchrist J, et al. Differential ranking of causes of fatal versus non-fatal injuries among US children. *Inj Prev* 2003;9(2):173-76. doi: 10.1136/ip.9.2.173

14. Ballesteros MF, Williams DD, Mack KA, et al. The epidemiology of unintentional and violence-related injury morbidity and mortality among children and adolescents in the United States. *Int J Environ Res Public Health* 2018;15(4) doi: 10.3390/ijerph15040616

15. Basco AN, McCormack ER, Basco WT. Age- and sex-related differences in nonfatal dog bite injuries among persons aged 0-19 treated in hospital emergency departments, United States, 2001-2017. *Public Health Rep* 2020;135(2):238-44. doi: 10.1177/0033354920904072

16. Bell TM, Qiao N, Jenkins PC, et al. Trends in emergency department visits for nonfatal violence-related injuries among adolescents in the United States, 2009-2013. *J Adolesc Health* 2016;58(5):573-75. doi: 10.1016/j.jadohealth.2015.12.016

17. Bergen G, Peterson C, Ederer D, et al. Vital signs: Health burden and medical costs of nonfatal injuries to motor vehicle occupants — United States, 2012. *MMWR Morb Mortal Wkly Rep* 2014;63(40):894-900.

18. Blindauer K, Gilchrist J, Ballesteros MF. Nonfatal residential fire-related injuries treated in emergency departments--United States, 2001. *MMWR Morb Mortal Wkly Rep* 2003;52(38):906-8. [published Online First: 2003/09/26]

19. Brenner RA, Taneja GS, Schroeder TJ, et al. Unintentional injuries among youth with developmental disabilities in the United States, 2006-2007. *Int J Inj Contr Saf Promot* 2013;20(3):259-65. doi: 10.1080/17457300.2012.696662 [published Online First: 2012/07/05]

20. Bridge JA, Horowitz LM, Fontanella CA, et al. Prioritizing research to reduce youth suicide and suicidal behavior. *Am J Prev Med* 2014;47(3):S229-S34.

21. Budnitz DS, Pollock DA, Weidenbach KN, et al. National surveillance of emergency department visits for outpatient adverse drug events. *JAMA* 2006;296(15):1858-66. doi: 10.1001/jama.296.15.1858

22. Burt A, Annest JL, Ballesteros MF, et al. Nonfatal, unintentional medication exposures among young children--United States, 2001-2003. *MMWR Morb Mortal Wkly Rep* 2006;55(1):1-5. [published Online First: 2006/01/18]

23. Centers for Disease Control and Prevention (CDC). National estimates of nonfatal injuries treated in hospital emergency departments--United States, 2000. *MMWR Morb Mortal Wkly Rep* 2001;50(17):340-6. [published Online First: 2001/07/24]

24. Centers for Disease Control and Prevention (CDC). Fall-related injuries during the holiday season--United States, 2000-2003. *MMWR Morb Mortal Wkly Rep* 2004;53(48):1127-9. [published Online First: 2004/12/14]

25. Chang DC, Williams M, Sangji NF, et al. Pattern of law enforcement-related injuries in the United States. *J Trauma Acute Care Surg* 2016;80(6):870-76. doi: 10.1097/TA.0000000000001000

26. Chang DT, Abdo K, Bhatt JM, et al. Persistence of choking injuries in children. *Int J Pediatr Otorhinolaryngol* 2021;144 doi: 10.1016/j.ijporl.2021.110685

27. Chang JT, Wang B, Rostron BL, et al. National estimates of ENDS liquid nicotine exposures, U.S., 2013–2017. *Am J Prev Med* 2020;59(5):742-45. doi: 10.1016/j.amepre.2020.05.027

28. Chapin MM, Rochette LM, Annest JL, et al. Nonfatal choking on food among children 14 years or younger in the united states, 2001-2009. *Pediatrics* 2013;132(2):275-81. doi: 10.1542/peds.2013-0260

29. Chen WS, Dunn RY, Chen AJ, et al. Epidemiology of nonfatal bicycle injuries presenting to United States emergency departments, 2001-2008. *Acad Emerg Med* 2013;20(6):570-75. doi: 10.1111/acem.12146

30. Cheng TA, Bell JM, Haileyesus T, et al. Nonfatal playground-related traumatic brain injuries among Children, 2001-2013. *Pediatrics* 2016;137(6) doi: 10.1542/peds.2015-2721

31. Claassen CA, Trivedi MH, Shimizu I, et al. Epidemiology of nonfatal deliberate self-harm in the United States as described in three medical databases. *Suicide Life Threat Behav* 2006;36(2):192-212. doi: 10.1521/suli.2006.36.2.192

32. Conn JM, Annest JL, Bossarte RM, et al. Non-fatal sports and recreational violent injuries among children and teenagers, United States, 2001-2003. *J Sci Med Sport* 2006;9(6):479-89. doi: 10.1016/j.jsams.2006.03.004

33. Conn JM, Annest JL, Dellinger A. Nonfatal motor-vehicle animal crash-related injuries--United States, 2001-2002. *MMWR Morb Mortal Wkly Rep* 2004;53(30):675-8. [published Online First: 2004/08/06]

34. Conn JM, Annest JL, Paulozzi LJ. Nonfatal injuries from off-road motorcycle riding among children and teens--United States, 2001-2004. *MMWR Morb Mortal Wkly Rep* 2006;55(22):621-4. [published Online First: 2006/06/09]

35. Conn JM, Annest JL, Ryan GW, et al. Non-work-related finger amputations in the United States, 2001-2002. *Ann Emerg Med* 2005;45(6):630-35. doi: 10.1016/j.annemergmed.2004.10.012

36. Coronado VG, Haileyesus T, Cheng TA, et al. Trends in sports- and recreation-related traumatic brain injuries treated in US emergency departments: The National Electronic Injury Surveillance System-All Injury Program (NEISS-AIP) 2001-2012. *J Head Trauma Rehabil* 2015;30(3):185-97. doi: 10.1097/HTR.0000000000000156 [published Online First: 2015/05/09]

37. Corso PS, Finkelstein E, Miller T, et al. Incidence and lifetime costs of injuries in the United States. *Inj Prev* 2006;12(4):212-18. doi: 10.1136/ip.2005.010983

38. Corso PS, Mercy JA, Simon TR, et al. Medical costs and productivity losses due to interpersonal and self-directed violence in the United States. *Am J Prev Med* 2007;32(6):474-82.e2. doi: 10.1016/j.amepre.2007.02.010

39. Crosby AE, Ryan G, Logan JE. Nonfatal self-inflicted injuries among adults aged > or = 65 years--United States, 2005. *MMWR Morb Mortal Wkly Rep* 2007;56(38):989-93. [published Online First: 2007/09/28]

40. David-Ferdon CF, Haileyesus T, Liu Y, et al. Nonfatal assaults among persons aged 10-24 years - United States, 2001-2015. *MMWR Morb Mortal Wkly Rep* 2018;67(5):141-45. doi: 10.15585/mmwr.mm6705a1 [published Online First: 2018/02/09]

41. Dellinger AM. Non-fatal transportation injuries among women: Differences in injury patterns and severity by age. *J Safety Res* 2005;36(2):203-06. doi: 10.1016/j.jsr.2005.02.002

42. Dellinger AM, Boyd RM, Haileyesus T. Fall injuries in older adults from an unusual source: Entering and exiting a vehicle. *J Am Geriatr Soc* 2008;56(4):609-14. doi: 10.1111/j.1532-5415.2008.01638.x

43. Dellinger AM, Gilchrist J. Leading causes of fatal and nonfatal unintentional injury for children and teens and the role of lifestyle clinicians. *Am J Lifestyle Med* 2019;13(1):7-21. doi: 10.1177/1559827617696297 [published Online First: 2017/08/29]

44. Elder RW, Shults RA, Swahn MH, et al. Alcohol-related emergency department visits among people ages 13 to 25 years. *J Stud Alcohol* 2004;65(3):297-300. doi: 10.15288/jsa.2004.65.297

45. Feldman JM, Chen JT, Waterman PD, et al. Temporal trends and racial/ethnic inequalities for legal intervention injuries treated in emergency departments: US men and women age 15-34, 2001-2014. *J Urban Health* 2016;93(5):797-807. doi: 10.1007/s11524-016-0076-3 [published Online First: 2016/09/09]

46. Florence C, Haegerich T, Simon T, et al. Estimated lifetime medical and work-loss costs of emergency department-treated nonfatal injuries--United States, 2013. *MMWR Morb Mortal Wkly Rep* 2015;64(38):1078-82. doi: 10.15585/mmwr.mm6438a5 [published Online First: 2015/10/01]

47. Flores AH, Haileyesus T, Greenspan AI. National estimates of outdoor recreational injuries treated in emergency departments, United States, 2004-2005. *Wilderness Environ Med* 2008;19(2):91-98. doi: 10.1580/07-WEME-OR-152.1

48. Friedmann H, Kohn R. Exponential lethality: upper limits on suicide risk assessments. *R I Med J* 2004;87(5):146.

49. Friedmann H, Kohn R. Mortality, or probability of death, from a suicidal act in the United States. *Suicide Life Threat Behav* 2008;38(3):287-301. doi: 10.1521/suli.2008.38.3.287

50. Gavin L, Mackay AP, Brown K, et al. Sexual and reproductive health of persons aged 10-24 Years - United States, 2002-2007. *MMWR Morb Mortal Wkly Rep* 2009;58(SS-6):1-58.

51. Gaw CE, Zonfrillo MR. Emergency department visits for head trauma in the United States. *BMC Emerg Med* 2016;16(1) doi: 10.1186/s12873-016-0071-8

52. Gerson LW, Stevens JA. Recreational injuries among older Americans, 2001. *Inj Prev* 2004;10(3):134-38. doi: 10.1136/ip.2004.005256

53. Gilchrist J, Gotsch K, Annest JL, et al. Nonfatal dog bite-related injuries treated in hospital emergency departments--United States, 2001. *MMWR Morb Mortal Wkly Rep* 2003;52(26):605-10. [published Online First: 2003/07/05]

54. Gilchrist J, Gotsch K, Ryan G. Nonfatal and fatal drownings in recreational water settings--United States, 2001-2002. *MMWR Morb Mortal Wkly Rep* 2004;53(21):447-52. [published Online First: 2004/06/04]

55. Gilchrist J, Haileyesus T, Murphy MW, et al. Nonfatal sports and recreation heat illness treated in hospital emergency departments --- United States, 2001--2009. *MMWR Morb Mortal Wkly Rep* 2011;60(29):977-80.

56. Gilchrist J, Thomas KE, Wald M, et al. Nonfatal traumatic brain injuries from sports and recreation activities--United States, 2001-2005. *MMWR Morb Mortal Wkly Rep* 2007;56(29):733-7. [published Online First: 2007/07/28]

57. Gilchrist J, Thomas KE, Xu L, et al. Nonfatal traumatic brain injuries related to sports and recreation activities among persons aged ≤19 years--United States, 2001-2009. *MMWR Morb Mortal Wkly Rep* 2011;60(39):1337-42. [published Online First: 2011/10/07]

58. Godoy Garraza L, Peart Boyce S, Walrath C, et al. An economic evaluation of the Garrett Lee Smith Memorial Suicide Prevention Program. *Suicide Life Threat Behav* 2018;48(1):3-11. doi: 10.1111/sltb.12321 [published Online First: 20161216]

59. Gotsch K, Annest JL, Holmgreen P, et al. Nonfatal choking-related episodes among children--United States, 2001. *MMWR Morb Mortal Wkly Rep* 2002;51(42):945-48.

60. Gotsch K, Annest JL, Holmgreen P, et al. Nonfatal sports- and recreation-related injuries treated in emergency departments--United States, July 2000-June 2001. *MMWR Morb Mortal Wkly Rep* 2002;51(33):736-40. [published Online First: 2002/08/31]

61. Guy GP, Jr., Watson M, Haileyesus T, et al. Indoor tanning-related injuries treated in a national sample of US hospital emergency departments. *JAMA Intern Med* 2015;175(2):309-11. doi: 10.1001/jamainternmed.2014.6697 [published Online First: 2014/12/17]

62. Haarbauer-Krupa J, Haileyesus T, Gilchrist J, et al. Fall-related traumatic brain injury in children ages 0-4years. *J Safety Res* 2019;70:127-33. doi: 10.1016/j.jsr.2019.06.003 [published Online First: 2019/12/19]

63. Haddad YK, Shakya I, Moreland BL, et al. Injury diagnosis and affected body part for nonfatal fall-related injuries in community-dwelling older adults treated in emergency departments. *J Aging Health* 2020 doi: 10.1177/0898264320932045

64. Haileyesus T, Annest JL, Dellinger AM. Cyclists injured while sharing the road with motor vehicles. *Inj Prev* 2007;13(3):202-06. doi: 10.1136/ip.2006.014019

65. Haileyesus T, Annest JL, Mercy JA. Non-fatal conductive energy device-related injuries treated in US emergency departments, 2005-2008. *Inj Prev* 2011;17(2):127-30. doi: 10.1136/ip.2010.028704

66. Hareza D, Langley R, Haskell MG, et al. National estimates of noncanine bite and sting injuries treated in US hospital emergency departments, 2011-2015. *South Med J* 2020;113(5):232-39. doi: 10.14423/SMJ.0000000000001091

67. Hartholt KA, Stevens JA, Polinder S, et al. Increase in fall-related hospitalizations in the United States, 2001-2008. *J Trauma* 2011;71(1):255-58. doi: 10.1097/TA.0b013e31821c36e7

68. Head EN, Stevens JA, Haileyesus T. Bathroom injuries in children less than 15 years old. *Inj Prev* 2013;19(5):316-19. doi: 10.1136/injuryprev-2012-040600

69. Hu G, Baker SP. Recent increases in fatal and non-fatal injury among people aged 65 years and over in the USA. *Inj Prev* 2010;16(1):26-30. doi: 10.1136/ip.2009.023481

70. Hungerford DW, Sullivent E, Thomas K, et al. Nonfatal scald-related burns among adults aged >/=65 years--United States, 2001-2006. *MMWR Morb Mortal Wkly Rep* 2009;58(36):993-6. [published Online First: 2009/09/19]

71. Ikeda R, Mahendra RR, Saltzman LE, et al. Nonfatal self-inflicted injuries treated in hospital emergency departments--United States, 2000. *MMWR Morb Mortal Wkly Rep* 2002;51(20):436-8. [published Online First: 2002/06/12]

72. Jackson TL, Mello MJ. Injury patterns and severity among motorcyclists treated in US emergency departments, 2001-2008: A comparison of younger and older riders. *Inj Prev* 2013;19(5):297-302. doi: 10.1136/injuryprev-2012-040619

73. Jason J. Community-acquired, non-occupational needlestick injuries treated in US Emergency Departments. *J Public Health (Oxf)* 2013;35(3):422-30. doi: 10.1093/pubmed/fdt033

74. Kakara RS, Moreland BL, Haddad YK, et al. Seasonal variation in fall-related emergency department visits by location of fall – United States, 2015. *J Safety Res* 2021 doi: https://doi.org/10.1016/j.jsr.2021.08.002

75. Kalesan B, Adhikarla C, Pressley JC, et al. The hidden epidemic of firearm injury: Increasing firearm injury rates during 2001-2013. *Am J Epidemiol* 2017;185(7):546-53. doi: 10.1093/aje/kww147

76. Kalesan B, Weinberg J, Galea S. Gun violence in Americans' social network during their lifetime. *Prev Med* 2016;93:53-56. doi: 10.1016/j.ypmed.2016.09.025

77. Kantar RS, Alfonso AR, Ramly EP, et al. Incidence of preventable nonfatal craniofacial injuries and implications for facial transplantation. *J Craniofac Surg* 2019;30(7):2023-25. doi: 10.1097/SCS.0000000000005715

78. Katsiyannis A, Whitford DK, Ennis RP. Firearm violence across the lifespan: relevance and theoretical impact on child and adolescent educational prospects. *J Child Fam Stud* 2018;27(6):1748-62. doi: 10.1007/s10826-018-1035-2

79. Khurana B, Hines DA, Johnson BA, et al. Injury patterns and associated demographics of intimate partner violence in men presenting to U.S. emergency departments. *Aggress Behav* 2021 doi: 10.1002/ab.22007

80. Khurana B, Loder RT. Injury patterns and associated demographics of intimate partner violence in older adults presenting to U.S. emergency departments. *J Interpers Violence* 2021:8862605211022060. doi: 10.1177/08862605211022060 [published Online First: 2021/06/15]

81. Khurana B, Raja A, Dyer GSM, et al. Upper extremity fractures due to intimate partner violence versus accidental causes. *Emerg Radiol* 2021 doi: 10.1007/s10140-021-01972-9

82. Kocher KE, Dellinger A. Public health and aging: nonfatal injuries among older adults treated in hospital emergency departments--United States, 2001. *MMWR Morb Mortal Wkly Rep* 2003;52(42):1019-22. [published Online First: 2003/10/24]

83. Kraemer JD. Epidemiology of non-fatal US emergency room visits for road crashes involving pedestrians in wheelchairs. *Inj Prev* 2015;21(5):331-4. doi: 10.1136/injuryprev-2014-041380 [published Online First: 2014/12/30]

84. Krajewski AK, Friedman LS. Hospital outcomes and economic costs from poisoning cases in Illinois. *Clin Toxicol (Phila)* 2015;53(5):433-45.

85. Langley R, Haskell MG, Hareza D, et al. Rodent bite injuries presenting to emergency departments in the united states, 2001–2015. *J Environ Health* 2021;83(7):18-25.

86. Langley R, Haskell MG, Hareza D, et al. Fatal and nonfatal snakebite injuries reported in the United States. *South Med J* 2020;113(10):514-19. doi: 10.14423/SMJ.0000000000001156

87. Langley R, Mack K, Haileyesus T, et al. National estimates of noncanine bite and sting injuries treated in US Hospital Emergency Departments, 2001-2010. *Wilderness Environ Med* 2014;25(1):14-23. doi: 10.1016/j.wem.2013.08.007 [published Online First: 2014/01/18]

88. Lawrence BA, Spicer RS, Miller TR. A fresh look at the costs of non-fatal consumer product injuries. *Inj Prev* 2015;21(1):23-9. doi: 10.1136/injuryprev-2014-041220 [published Online First: 2014/08/03]

89. Linakis JG, Amanullah S, Mello MJ. Emergency department visits for injury in school‐aged children in the United States: a comparison of nonfatal injuries occurring within and outside of the school environment. *Acad Emerg Med* 2006;13(5):567-70.

90. Loder RT. The demographics of dog bites in the United States. *Heliyon* 2019;5(3) doi: 10.1016/j.heliyon.2019.e01360

91. Loder RT, Leiser A. Injury patterns and demographics due to legal intervention seen in US emergency departments. *J Forensic Leg Med* 2021;79:102150. doi: 10.1016/j.jflm.2021.102150 [published Online First: 2021/03/14]

92. Loder RT, Meixner C. The demographics of dog bites due to K-9 (legal intervention) in the United States. *J Forensic Leg Med* 2019;65:9-14. doi: 10.1016/j.jflm.2019.04.008

93. Loder RT, Momper L. Demographics and fracture patterns of patients presenting to US emergency departments for intimate partner violence. *J Am Acad Orthop Surg Glob Res Rev* 2020;4(2) doi: 10.5435/JAAOSGlobal-D-20-00009

94. Loder RT, Palma S, Smith M. Injury patterns and demographics in child and adolescent assault victims presenting to US emergency departments. *Int J Pediatr* 2020;2020 doi: 10.1155/2020/8169030

95. Loder RT, Robinson TP. The demographics of patients presenting for sexual assault to US emergency departments. *J Forensic Leg Med* 2020;69 doi: 10.1016/j.jflm.2019.101887

96. Logan JE, Haileyesus T, Ertl A, et al. Nonfatal assaults and homicides among adults aged >/=60 Years - United States, 2002-2016. *MMWR Morb Mortal Wkly Rep* 2019;68(13):297-302. doi: 10.15585/mmwr.mm6813a1 [published Online First: 2019/04/05]

97. Mack KA. Fatal and nonfatal unintentional injuries in adult women, United States. *J Womens Health (Larchmt)* 2004;13(7):754-62. doi: 10.1089/jwh.2004.13.754

98. Mack KA, Gilchrist J, Ballesteros MF. Bunk bed-related injuries sustained by young children treated in emergency departments in the United States, 2001-2004, National Electronic Injury Surveillance System - All injury program. *Inj Prev* 2007;13(2):137-40. doi: 10.1136/ip.2006.013193

99. Mack KA, Gilchrist J, Ballesteros MF. Unintentional injuries among infants age 0-12 months. *J Safety Res* 2007;38(5):609-12. doi: 10.1016/j.jsr.2007.08.001

100. Mack KA, Gilchrist J, Ballesteros MF. Injuries among infants treated in emergency departments in the United States, 2001-2004. *Pediatrics* 2008;121(5):930-37. doi: 10.1542/peds.2007-1731

101. Marlenga B, Berg RL, Pickett W. National public dealth data systems in the United States: applications to child agricultural injury surveillance. *J Rural Health* 2018;34(3):314-21. doi: 10.1111/jrh.12292

102. Marsh SM, Reichard AA, Bhandari R, et al. Using emergency department surveillance data to assess occupational injury and illness reporting by workers. *Am J Ind Med* 2016;59(8):600-9. doi: 10.1002/ajim.22615 [published Online First: 2016/07/12]

103. McGeehan J, Annest JL, Vajani M, et al. School bus-related injuries among children and teenagers in the United States, 2001-2003. *Pediatrics* 2006;118(5):1978-84. doi: 10.1542/peds.2006-1314

104. McGwin Jr G, Owsley C. Incidence of emergency department-treated eye injury in the United States. *Arch Ophthalmol* 2005;123(5):662-66. doi: 10.1001/archopht.123.5.662

105. Meixner C, Loder RT. The demographics of fractures and dislocations across the entire United States due to common sports and recreational activities. *Sports Health* 2020;12(2):159-69. doi: 10.1177/1941738119882930

106. Mello MJ, Linakis J, Meyer S, et al. Bicycle related traumatic brain injury at Hasbro Children's Hospital: 1997-2003. *R I Med J* 2005;88(6):192.

107. Mello MJ, Myers R, Christian JB, et al. Injuries in youth football: National emergency department visits during 2001-2005 for young and adolescent players. *Acad Emerg Med* 2009;16(3):243-48. doi: 10.1111/j.1553-2712.2009.00357.x

108. Mercado MC, Holland K, Leemis RW, et al. Trends in emergency department visits for nonfatal self-inflicted injuries among youth aged 10 to 24 Years in the United States, 2001-2015. *JAMA* 2017;318(19):1931-33. doi: 10.1001/jama.2017.13317 [published Online First: 2017/11/23]

109. Mitchell RA, Hasbrouck L, Ingram E, et al. Nonfatal physical assault-related injuries among persons aged ≥60 years treated in hospital emergency departments - United States, 2001. *MMWR Morb Mortal Wkly Rep* 2003;52(34):812-16.

110. Mitchell RA, Hasbrouck L, Ingram E, et al. Public health and aging: nonfatal physical assault-related injuries among persons aged >60 years treated in hospital emergency departments--United States, 2001. *MMWR Morb Mortal Wkly Rep* 2003;52(34):812-6.

111. Monuteaux MC, Azrael D, Miller M. Association of increased safe household firearm storage with firearm suicide and unintentional death among US youths. *JAMA Pediatr* 2019;173(7):657-62.

112. Moreland BL, Kakara R, Haddad YK, et al. A descriptive analysis of location of older adult falls that resulted in emergency department visits in the United States, 2015. *Am J Lifestyle Med* 2020 doi: 10.1177/1559827620942187

113. Naumann RB, Dellinger AM, Haileyesus T, et al. Older adult pedestrian injuries in the United States: Causes and contributing circumstances. *Int J Inj Contr Saf Promot* 2011;18(1):65-73. doi: 10.1080/17457300.2010.517321

114. Naumann RB, Dellinger AM, Zaloshnja E, et al. Incidence and total lifetime costs of motor vehicle - related fatal and nonfatal injury by road user type, United States, 2005. *Traffic Inj Prev* 2010;11(4):353-60. doi: 10.1080/15389588.2010.486429

115. Noonan RK, Stevens JA, Baldwin G. The public health approach to older adult fall prevention: Comments from the US Centers for Disease Control and Prevention. *Open Longev Sci* 2011;5(1)

116. Nourjah P, Ahmad SR, Karwoski C, et al. Estimates of acetaminophen (paracetamol)-associated overdoses in the United States. *Pharmacoepidemiol Drug Saf* 2006;15(6):398-405. doi: 10.1002/pds.1191

117. O'Neil ME, Mack KA, Gilchrist J. Epidemiology of non-canine bite and sting injuries treated in U.S. Emergency Departments, 2001-2004. *Public Health Rep* 2007;122(6):764-75. doi: 10.1177/003335490712200608

118. O'Neil ME, Mack KA, Gilchrist J, et al. Snakebite injuries treated in United States emergency departments, 2001-2004. *Wilderness Environ Med* 2007;18(4):281-87. doi: 10.1580/06-WEME-OR-080R1.1

119. Olufajo OA, Williams M, Ahuja G, et al. Patterns and trends of gun violence against women in the United States. *Ann Surg* 2021;273(6):1115-19. doi: 10.1097/SLA.0000000000004810

120. Orces CH. Emergency department visits for fall-related fractures among older adults in the USA: A Retrospective cross-sectional analysis of the National Electronic Injury Surveillance System All Injury Program, 2001-2008. *BMJ Open* 2013;3(1) doi: 10.1136/bmjopen-2012-001722

121. Orces CH, Alamgir H. Trends in fall-related injuries among older adults treated in emergency departments in the USA. *Inj Prev* 2014

122. Orces CH, Martinez FJ. Epidemiology of fall related forearm and wrist fractures among adults treated in US hospital emergency departments. *Inj Prev* 2011;17(1):33-36. doi: 10.1136/ip.2010.026799

123. Orces CH, Orces J. Trends in the U.S. childhood emergency department visits for fall-related fractures, 2001-2015. *Cureus* 2020;12(11):e11629. doi: 10.7759/cureus.11629 [published Online First: 2020/12/31]

124. Owens PL, Barrett ML, Gibson TB, et al. Emergency department care in the United States: a profile of national data sources. *Ann Emerg Med* 2010;56(2):150-65.

125. Patel R, Dellinger A, Annest JL. Nonfatal motor-vehicle-related backover injuries among children--United States, 2001-2003. *MMWR Morb Mortal Wkly Rep* 2005;54(6):144-6. [published Online First: 2005/02/18]

126. Patel R, Dellinger AM. Motor-vehicle boarding and alighting injury—how large a problem? *J Safety Res* 2006;37(3):321-23.

127. Peterson C, Foster SL, Xu L, et al. US campus fraternities and sororities and the young adult injury burden. *J Am Coll Health* 2018;66(5):340-49. doi: 10.1080/07448481.2018.1431899

128. Peterson C, Miller GF, Barnett SBL, et al. Economic cost of injury - United States, 2019. *MMWR Morb Mortal Wkly Rep* 2021;70(48):1655-59. doi: 10.15585/mmwr.mm7048a1 [published Online First: 20211203]

129. Prosser JM, Perrone J, Pines JM. The epidemiology of intentional non-fatal self-harm poisoning in the United States: 2001-2004. *J Med Toxicol* 2007;3(1):20-24. doi: 10.1007/BF03161034

130. Purtle J, Rich LJ, Bloom SL, et al. Cost− benefit analysis simulation of a hospital-based violence intervention program. *Am J Prev Med* 2015;48(2):162-69.

131. Quinlan KP, Annest JL, Myers B, et al. Neck strains and sprains among motor vehicle occupants - United States, 2000. *Accid Anal Prev* 2004;36(1):21-27. doi: 10.1016/S0001-4575(02)00110-0

132. Rosen T, Mack KA, Noonan RK. Slipping and tripping: fall injuries in adults associated with rugs and carpets. *J Inj Violence Res* 2013;5(1):61-9. doi: 10.5249/jivr.v5i1.177 [published Online First: 2012/08/08]

133. Saltzman LE, Basile KC, Mahendra RR, et al. National estimates of sexual violence treated in emergency departments. *Ann Emerg Med* 2007;49(2):210-17. doi: 10.1016/j.annemergmed.2006.10.015

134. Saltzman LE, Mahendra RR, Ikeda RM, et al. Utility of hospital emergency department data for studying intimate partner violence. *J Marriage Fam* 2005;67(4):960-70. doi: 10.1111/j.1741-3737.2005.00187.x

135. Sanchez CA, Thomas KE, Malilay J, et al. Nonfatal natural and environmental injuries treated in emergency departments, united states, 2001-2004. *Fam Community Health* 2010;33(1):3-10. doi: 10.1097/FCH.0b013e3181c4e2fa

136. Sarmiento K, Haileyesus T, Waltzman D, et al. Emergency department visits for bicycle-related traumatic brain injuries among children and adults - United States, 2009-2018. *MMWR Morb Mortal Wkly Rep* 2021;70(19):693-97. doi: 10.15585/mmwr.mm7019a1 [published Online First: 2021/05/15]

137. Sarmiento K, Thomas KE, Daugherty J, et al. Emergency department visits for sports- and recreation-related traumatic brain injuries among children - United States, 2010-2016. *MMWR Morb Mortal Wkly Rep* 2019;68(10):237-42. doi: 10.15585/mmwr.mm6810a2 [published Online First: 2019/03/15]

138. Schillie SF, Shehab N, Thomas KE, et al. Medication overdoses leading to emergency department visits among children. *Am J Prev Med* 2009;37(3):181-87. doi: 10.1016/j.amepre.2009.05.018

139. Shakya I, Bergen G, Haddad YK, et al. Fall-related emergency department visits involving alcohol among older adults. *J Safety Res* 2020;74:125-31. doi: 10.1016/j.jsr.2020.06.001

140. Shields W, McDonald E, Frattaroli S, et al. Structural housing elements associated with home injuries in children. *Inj Prev* 2016;22(2):105-9. doi: 10.1136/injuryprev-2015-041621 [published Online First: 2015/08/22]

141. Shults RA, Elder RW, Hungerford DW, et al. Emergency department visits for alcohol-related unintentional traumatic injuries, United States, 2001. *J Safety Res* 2009;40(4):329-31.

142. Shults RA, West BA, Rudd RA, et al. All-terrain vehicle-related nonfatal injuries among young riders in the united states, 2001-2010. *Pediatrics* 2013;132(2):282-89. doi: 10.1542/peds.2013-0751

143. Shults RA, Wiles SD, Vajani M, et al. All-terrain vehicle-related nonfatal injuries among young riders: United States, 2001-2003. *Pediatrics* 2005;116(5):e608-e12. doi: 10.1542/peds.2005-0937

144. Simon T, Saltzman LE, Swahn MH, et al. Nonfatal physical assault-related injuries treated in hospital emergency departments--United States, 2000. *MMWR Morb Mortal Wkly Rep* 2002;51(21):460-3. [published Online First: 2002/06/11]

145. Skarbek-Borowska S, Amanullah S, Mello MJ, et al. Emergency department visits for sledding injuries in children in the United States in 2001/2002. *Acad Emerg Med* 2006;13(2):181-85. doi: 10.1197/j.aem.2005.09.009

146. Sleet DA, Liller KD, White DD, et al. Injuries, injury prevention and public health. *Am J Health Behav* 2004;28(1):S6-S12.

147. Sritharen Y, Hernandez MC, Zielinski MD, et al. Weekend woodsmen: overview and comparison of injury patterns associated with power saw and axe utilization in the United States. *Am J Emerg Med* 2018;36(5):846-50. doi: 10.1016/j.ajem.2018.01.047

148. Stevens JA, Corso PS, Finkelstein EA, et al. The costs of fatal and non-fatal falls among older adults. *Inj Prev* 2006;12(5):290-95. doi: 10.1136/ip.2005.011015

149. Stevens JA, Haas EN, Haileyesus T. Nonfatal bathroom injuries among persons aged ≥15 years--United States, 2008. *MMWR Morb Mortal Wkly Rep* 2011;60(22):729-33. [published Online First: 2011/06/11]

150. Stevens JA, Sogolow ED. Gender differences for non-fatal unintentional fall related injuries among older adults. *Inj Prev* 2005;11(2):115-19. doi: 10.1136/ip.2004.005835

151. Stevens JA, Teh SL. Nonfatal fall-related injuries associated with dogs and cats--United States, 2001-2006. *MMWR Morb Mortal Wkly Rep* 2009;58(11):277-81. [published Online First: 2009/03/28]

152. Stevens JA, Teh SL, Haileyesus T. Dogs and cats as environmental fall hazards. *J Safety Res* 2010;41(1):69-73. doi: 10.1016/j.jsr.2010.01.001

153. Stevens JA, Thomas K, Teh L, et al. Unintentional fall injuries associated with walkers and canes in older adults treated in U.S. emergency departments. *J Am Geriatr Soc* 2009;57(8):1464-69. doi: 10.1111/j.1532-5415.2009.02365.x

154. Stevens JA, Thomas KE, Sogolow ED. Seasonal patterns of fatal and nonfatal falls among older adults in the U.S. *Accid Anal Prev* 2007;39(6):1239-44. doi: 10.1016/j.aap.2007.03.011

155. Sumner SA, Mercy JA, Dahlberg LL, et al. Violence in the United States: status, challenges, and opportunities. *JAMA* 2015;314(5):478-88. doi: 10.1001/jama.2015.8371

156. Thiels CA, Hernandez MC, Zielinski MD, et al. Injury patterns and outcomes of ice-fishing in the United States. *Am J Emerg Med* 2016;34(7):1258-61. doi: 10.1016/j.ajem.2016.02.078 [published Online First: 2016/04/28]

157. Thomas KE, Annest JL, Gilchrist J, et al. Non-fatal horse related injuries treated in emergency departments in the United States. *Br J Sports Med* 2006;40(7):619-26. doi: 10.1136/bjsm.2006.025858

158. Thombs BD, Bresnick MG, Magyar-Russell G. Who attempts suicide by burning? An analysis of age patterns of mortality by self-inflicted burning in the United States. *Gen Hosp Psychiatry* 2007;29(3):244-50. doi: 10.1016/j.genhosppsych.2007.01.012

159. Tuckel PS, Milczarski W. The changing epidemiology of dog bite injuries in the United States, 2005-2018. *Inj Epidemiol* 2020;7(1):57. doi: 10.1186/s40621-020-00281-y [published Online First: 2020/11/02]

160. Vajani M, Annest JL, Ballesteros MF, et al. Unintentional non-fire-related carbon monoxide exposures--United States, 2001-2003. *MMWR Morb Mortal Wkly Rep* 2005;54(2):36-9. [published Online First: 2005/01/22]

161. Vajani M, Annest JL, Crosby AE, et al. Nonfatal and fatal self-harm injuries among children aged 10-14 years - United States and Oregon, 2001-2003. *Suicide Life Threat Behav* 2007;37(5):493-506. doi: 10.1521/suli.2007.37.5.493

162. Vyrostek SB, Annest JL, Ryan GW. Surveillance for fatal and nonfatal injuries--United States, 2001. *MMWR Surveill Summ* 2004;53(7):1-57.

163. Waltzman D, Womack LS, Thomas KE, et al. Trends in emergency department visits for contact sports-related traumatic brain injuries among children - United States, 2001-2018. *MMWR Morb Mortal Wkly Rep* 2020;69(27):870-74. doi: 10.15585/mmwr.mm6927a4 [published Online First: 2020/07/10]

164. Wei F, Hester AL. Gender difference in falls among adults treated in emergency departments and outpatient clinics. *J Gerontol Geriatr Res* 2014;3:152. doi: 10.4172/2167-7182.1000152 [published Online First: 2014/10/14]

165. Zagel AL, Cutler GJ, Linabery AM, et al. Unintentional injuries in primary and secondary schools in the United States, 2001-2013. *J Sch Health* 2019;89(1):38-47. doi: 10.1111/josh.12711

166. Zagel AL, Kreykes NS, Handt EA. Pediatric farm injuries presenting to United States emergency departments, 2001-2014. *J Rural Health* 2019;35(4):442-52. doi: 10.1111/jrh.12371

167. Zaloshnja E, Miller TR, Lawrence BA, et al. The costs of unintentional home injuries. *Am J Prev Med* 2005;28(1):88-94.
